# Supplementary material for: Filling gaps in bacterial catabolic pathways with computation and high-throughput genetics
Source: PLoS Genet. 2022 Apr 13;18(4):e1010156. doi: 10.1371/journal.pgen.1010156 (PMC9007349; doi:10.1371/journal.pgen.1010156)
Supplement: S1 Fig — (PDF) [file pgen.1010156.s001.pdf]

Arginine metabolism in *Pseudomonas fluorescens* FW300-N2E3

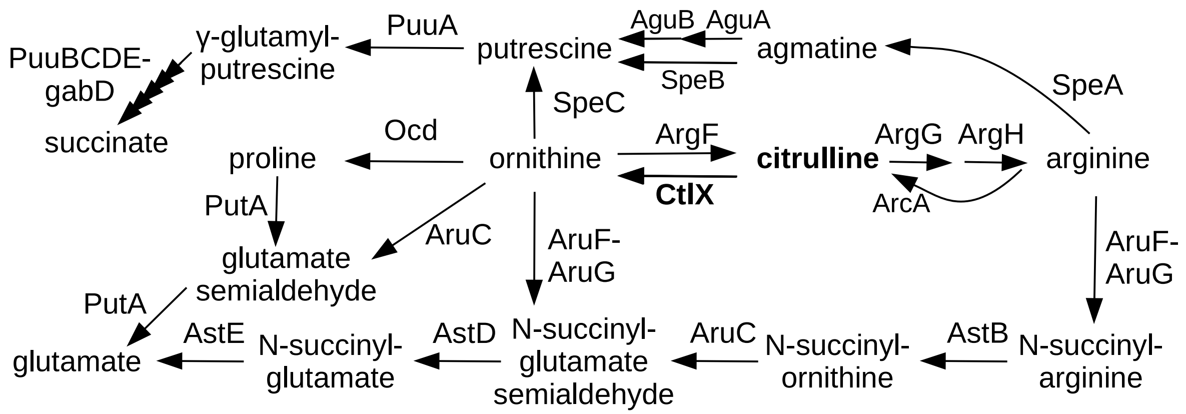

Gene fitness

Gene	Description	L-ornithine				L-citrulline						L-arginine				proline		D-glucose			
		10 mM		5 mM		10 mM		5 mM		20	5N	10 mM		2.5 mM		20 mM		10 mM		20 mM	
AO353_25635	putative citrullinase CtlX	-1.6	-2.3	-1.6	-1.9	-3.5	-3.6	-3.4	-2.2	-4.7	-5.8	-0.1	-0.1	0.1	0.0	0.3	-0.1	-0.3	0.4	-0.1	0.1
Succinylated intermediates																					
AO353_03015	arginine/ornithine succinyltransferase, AruF subunit	-1.6	-2.0	-2.3	-0.8	-1.7	-2.1	-1.1	-2.0	-3.2	-3.5	-3.4	-3.2	-3.9	-2.4	0.2	-0.2	-0.7	-1.0	-1.3	-1.5
AO353_03020	arginine/ornithine succinyltransferase, AruG subunit	0.4	2.1	-0.2	-0.4	-1.3	-1.3	-1.2	-1.1	-0.4	-1.8	-2.7	-2.2	-1.5	-1.2	-0.4	-0.3	-1.8	-1.3	-2.2	-1.8
AO353_25630	AruG-2	-3.2	-3.0	-3.5	-3.0	-3.0	-3.7	-3.1	-3.1	-4.4	-3.3	-0.3	-0.1	-0.1	0.1	-0.2	0.2	-0.2	-0.0	-0.2	0.2
AO353_03005	N-succinylarginine dihydrolase AstB	3.2	4.6	1.1	1.0	-1.9	-1.7	-1.1	-1.2	-4.3	-1.5	-3.1	-3.6	-4.5	-3.4	-0.0	0.1	0.2	0.0	-0.2	0.0
AO353_03025	(succinyl)ornithine 5-aminotransferase AruC	-2.7	-2.2	-3.0	-3.0	-2.7	-2.7	-2.5	-2.4	-4.3	-3.9	-3.7	-3.8	-4.0	-4.1	-0.3	-0.1	-0.3	-0.3	-0.7	-0.9
AO353_03010	succinylglutamate-semialdehyde dehydrogenase AstD	-2.1	-2.4	-2.7	-2.2	-1.5	-2.0	-1.8	-3.2	-3.5	-3.2	-3.2	-3.6	-3.1	-2.8	0.0	0.2	0.5	0.9	0.0	-0.1
AO353_02995	succinylglutamate desuccinylase AstE	-4.4	-3.1	-4.6	-4.2	-4.9	-4.3	-4.1	-4.5	-4.6	-5.0	-4.5	-4.7	-4.2	-4.5	0.1	-0.0	0.9	0.8	-0.2	-0.0
Arginine biosynthesis																					
AO353_04155	ornithine carbamoyltransferase ArgF	-2.3	-1.9	-2.0	-2.5	-0.3	-0.5	-0.3	-0.2	0.0	-0.1	0.0	-0.1	-0.4	-0.3	-3.1	-2.3	-2.3	-2.3	-3.0	-3.1
AO353_04105	argininosuccinate synthase ArgG	-2.1	-1.4	-2.2	-2.1	-2.0	-2.0	-1.8	-1.9	-2.8	-3.4	-0.2	-0.2	0.2	-0.1	-2.6	-2.4	-2.1	-2.1	-3.1	-3.3
AO353_09000	argininosuccinate lyase ArgH	-1.0	-0.4	-0.5	-1.5	-1.4	-1.0	-1.0	-0.7	-3.2	-3.1	-1.7	-1.5	-0.4	-0.3	-2.6	-2.2	-1.2	-0.1	-3.0	-3.4
AO353_25585	argininosuccinate lyase ArgH-2	-0.8	1.4	-0.6	-0.4	-0.5	-0.6	0.4	0.1	0.1	-0.2	1.1	0.4	-0.0	0.4	0.6	0.7	0.1	-0.2	0.9	1.1
Arginine decarboxylase pathway																					
AO353_13720	arginine decarboxylase SpeA	3.4	4.7	2.1	2.3	-0.1	-0.3	-0.2	-0.3	0.1	-0.1	-2.2	-2.0	-1.0	-1.1	-0.0	0.1	-0.2	-0.3	0.1	0.0
AO353_18400	agmatinase SpeB	1.5	1.8	0.5	1.2	0.1	-0.7	-0.4	-0.5	-0.5	-0.4	-0.4	-0.6	-0.2	-0.4	0.3	0.1	-0.5	-0.5	-0.1	0.0
AO353_11915	agmatine deiminase AguA	2.4	2.4	1.7	1.4	0.0	-0.0	-0.7	0.0	0.4	-0.0	-1.9	-1.9	-1.3	-0.8	0.3	-0.1	-0.1	-1.1	-0.3	-0.2
AO353_11910	N-carbamoylputrescine amidase AguB	1.0	1.2	-0.2	1.1	-0.1	0.2	-0.4	0.7	0.4	-0.0	-0.3	-0.6	-0.2	-0.1	0.2	-0.0	0.1	0.2	-0.2	-0.1
AO353_08595	γ-glutamyl-γ-putrescine synthetase PuuA	3.5	4.6	1.7	2.3	0.4	0.5	0.0	0.0	-1.8	-0.1	-3.1	-2.8	0.1	0.3	0.1	0.1	0.2	0.1	0.0	-0.1
Proline pathway																					
AO353_25185	ornithine cyclodeaminase Ocd	-1.6	-1.5	-0.9	-1.4	0.7	0.8	0.3	0.7	1.0	0.5	-0.0	0.1	0.1	-0.0	0.1	-0.2	0.2	0.3	0.2	-0.1
AO353_12810	proline dehydrogenase / 1-pyrroline-5-carboxylate dehydrogenase PutA	-1.8	-1.1	-1.8	-1.6	-0.7	-0.7	-0.8	-0.5	-0.9	-0.3	0.1	0.1	0.1	-0.0	-3.9	-3.2	0.0	0.1	-0.0	-0.0
Arginine deiminase																					
AO353_03470	arginine deiminase ArcA	2.5	3.5	1.0	1.0	0.1	0.2	1.0	-0.4	-0.2	0.2	0.0	-0.3	-0.5	-0.1	0.0	-0.1	-0.4	-0.3	-0.3	-0.1

Supplementary Figure 1: Utilization of arginine, citrulline, ornithine, and proline by *Pseudomonas fluorescens* FW300-N2E3. The top panel shows the potential pathways that are present in the genome. The heatmap shows fitness data for those genes. *ArcB*, *arcC* (carbamate kinase) and *speA* had little phenotype and are not shown. *P. fluorescens* was grown in a defined minimal medium with vitamins, minerals, 0.25 g/L ammonium chloride, and the indicated carbon source.
