# Supplementary material for: Filling gaps in bacterial catabolic pathways with computation and high-throughput genetics
Source: PLoS Genet. 2022 Apr 13;18(4):e1010156. doi: 10.1371/journal.pgen.1010156 (PMC9007349; doi:10.1371/journal.pgen.1010156)
Supplement: S2 Fig — (PDF) [file pgen.1010156.s002.pdf]

Arginine metabolism in *Pseudomonas simiae* WCS417

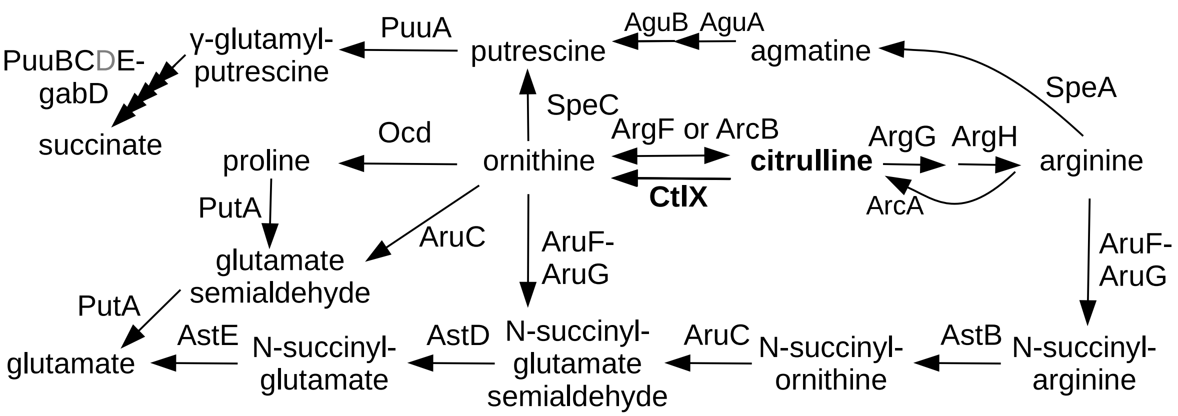

Gene fitness

Gene	Description	L-ornithine 10 mM		L-ornithine 5 mM		L-citrulline 10 mM		L-citrulline 5 mM		L-citrulline 20 mM		arginine 20 mM		D-glucose 20 mM		
PS417_17580	putative citrullinase CtlX	-5.5	-2.5	-3.3	-2.6	-3.6	-3.6	-3.8	-3.6	-4.8	-3.5	-0.4	-0.1	-0.0	-0.1	0.2
Proline pathway																
PS417_17585	ornithine cyclodeaminase Ocd	-3.6	-2.1	-2.8	-3.4	-2.7	-3.8	-3.5	-3.5	-2.9	-2.5	0.3	0.1	0.2	0.2	-0.2
PS417_02175	proline dehydrogenase / 1-pyrroline-5-carboxylate dehydrogenase PutA	-2.8	-2.3	-3.6	-2.8	-3.8	-2.5	-3.0	-4.4	-5.1	-2.7	-0.4	0.2	-0.1	0.2	0.4
Succinylated intermediates																
PS417_21705	arginine/ornithine succinyltransferase, AruF subunit	0.8	0.3	0.4	-0.0	0.1	-0.4	-0.2	-0.2	-0.4	-0.5	-2.6	-2.4	-2.4	-2.4	-1.7
PS417_21700	arginine/ornithine succinyltransferase, AruG subunit	-1.0	-0.9	-1.0	-1.5	-0.7	-0.6	-1.0	-1.0	-0.6	-0.7	-4.0	-5.5	-1.5	-1.1	-1.3
PS417_21690	N-succinylarginine dihydrolase AstB	0.3	0.2	-0.0	-0.1	-0.5	-1.0	-1.0	-1.0	-0.9	-1.2	-5.3	-6.1	-1.1	-1.0	-0.9
PS417_21710	(succinyl)ornithine 5-aminotransferase AruC	-1.9	-1.1	-0.6	-1.9	-0.9	-0.6	-0.8	-0.6	-0.4	-0.8	-4.1	-3.6	0.1	-0.2	0.1
PS417_21695	succinylglutamate-semialdehyde dehydrogenase AstD	-1.3	-1.4	-1.8	-1.3	-1.0	-0.8	-0.5	-0.4	-0.7	-0.4	-1.3	-2.8	-0.5	-0.4	-0.3
PS417_21680	succinylglutamate desuccinylase AstE	-1.3	-1.9	-1.4	-2.0	-1.5	-1.1	-1.8	-1.0	-0.6	-0.8	-3.6	-3.1	0.0	-0.0	0.4
Arginine biosynthesis																
PS417_05595	ornithine carbamoyltransferase ArgF	-2.9	-1.9	-2.5	-2.5	0.1	0.3	0.2	0.1	-0.1	-0.0	-0.4	-0.4	-3.6	-3.7	-3.9

Supplementary Figure 2: Utilization of arginine, citrulline, and ornithine by *Pseudomonas simiae* WCS417. The top panel shows the potential pathways that are present in the genome. The heatmap shows fitness data for those genes. *ArgG* and *argH* are not shown due to insufficient coverage to estimate fitness values. *ArcAB*, *arcC* (carbamate kinase), *speA*, *aguAB*, *puuBCE-gabD*, *aguB*, and *speC* had little phenotype under these conditions and are not shown. *PuuA* was important for arginine utilization but not for ornithine or citrulline utilization and is not shown. *PuuABCE* were very important for putrescine utilization, which confirms that they are annotated correctly, but we did not find the γ-glutamyl-γ-aminobutyrate putrescine hydrolase *puuD* in the genome. *P. simiae* was grown in a defined minimal medium with vitamins, minerals, 0.25 g/L ammonium chloride, and the indicated carbon source.
