# Supplementary material for: Filling gaps in bacterial catabolic pathways with computation and high-throughput genetics
Source: PLoS Genet. 2022 Apr 13;18(4):e1010156. doi: 10.1371/journal.pgen.1010156 (PMC9007349; doi:10.1371/journal.pgen.1010156)
Supplement: S3 Fig — (PDF) [file pgen.1010156.s003.pdf]

Arginine metabolism in *Phaeobacter inhibens* BS107

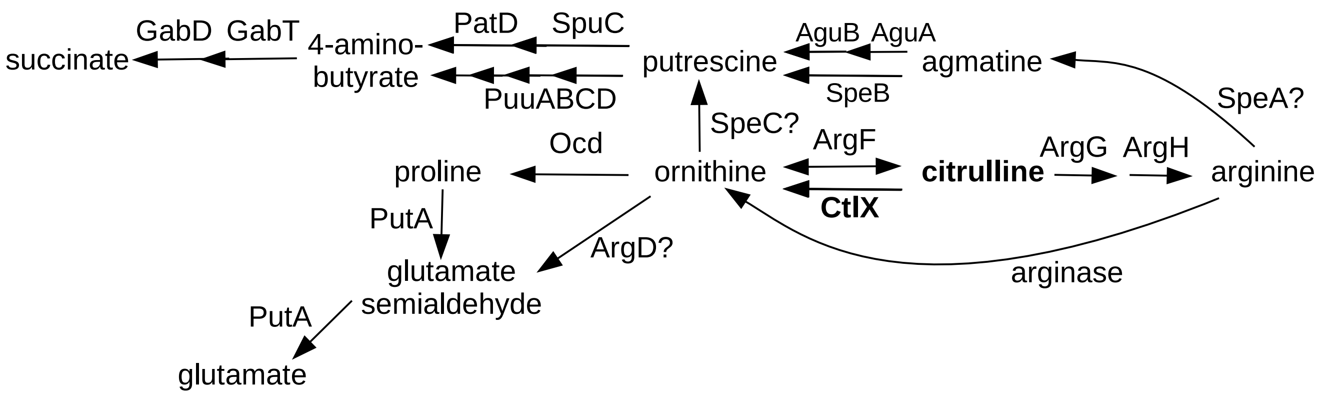

Gene fitness

Gene	Description	L-ornithine				L-citrulline								L-proline				D-glucose					
		10 mM		5 mM		10 mM		5 mM		20 mM		20 mM N		20 mM				10 mM		20 mM			
PGA1_c16380	putative citrullinase CtlX	-2.2	-2.2	-1.2	-1.2	-2.4	-2.1	-2.6	-4.1	-4.2	-4.8	-2.0	-2.3	0.5	0.1	0.1	0.1	0.1	-0.2	-0.4	-0.5	-0.3	-0.1
Proline pathway																							
PGA1_c16390	ornithine cyclodeaminase Ocd	-3.8	-2.7	-1.7	-0.4	-2.2	-1.7	-3.0	-3.0	-5.1	-3.4	-1.6	-3.2	0.0	0.2	0.3	0.0	-0.3	-0.7	0.2	-0.3	0.4	-0.1
PGA1_c11750	proline dehydrogenase / 1-pyrroline-5-carboxylate dehydrogenase PutA	-3.7	-2.8	-2.4	-1.8	-2.6	-2.7	-3.3	-3.5	-3.8	-4.0	-1.1	-1.1	-4.1	-5.0	-1.9	-1.6	-0.0	-0.3	-0.5	0.4	0.0	0.2
Arginine biosynthesis																							
PGA1_c24230	acetylornithine / ornithine (?) / N-succinyl-DAP aminotransferase ArgD	-1.2	-0.7	-1.9	-1.5	-1.7	-1.6	-1.4	-1.9	-0.8	-0.3	-0.1	-0.1	-3.4	-4.2	-2.0	-1.3	-2.0	-2.7	-3.1	-2.9	-4.3	-4.3
PGA1_c24220	ornithine carbamoyltransferase ArgF	-3.1	-2.5	-1.7	-1.3	-1.1	-1.2	-1.1	-1.0	0.4	-0.8	0.1	-0.8	-3.2	-3.4	-3.3	-0.8	-2.8	-2.8	-2.8	-3.2	-4.5	-4.2
Other																							
PGA1_c16370	arginase	-2.6	-3.2	-1.3	-4.7	-2.5	-1.8	-3.9	-3.2	-3.9	-3.3	-1.9	-2.1	-0.0	-0.0	0.2	0.0	-0.4	-0.4	0.6	0.1	0.4	0.2

Supplementary Figure 3: Utilization of citrulline, ornithine, and proline by *Phaeobacter inhibens* DSM 17395 (BS107). The top panel shows the potential pathways that are present in the genome. The heatmap shows fitness data for those genes. *ArgG* and *argH* are not shown due to insufficient coverage to estimate fitness values. *PGA1_c03800* (*speA* or *speC?*), *speB*, *aguAB*, *spuC*, *patD*, *puuABCD*, *gabT*, and *gabD* had little phenotype in these conditions and are not shown. *P. inhibens* was grown in a defined minimal medium with vitamins, minerals, and 1 g/L ammonium sulfate, except for experiments with citrulline as the sole source of N, for which 20 mM sucrose was added and ammonium sulfate was omitted.
