# Supplementary material for: Filling gaps in bacterial catabolic pathways with computation and high-throughput genetics
Source: PLoS Genet. 2022 Apr 13;18(4):e1010156. doi: 10.1371/journal.pgen.1010156 (PMC9007349; doi:10.1371/journal.pgen.1010156)
Supplement: Attachment — Submitted filename: GapMind_carbon_response.pdf [file pgen.1010156.s004.pdf]

Our changes

1. To focus the manuscript on how combining GapMind with fitness data helps to discover new biology, we revised the title, abstract, and Introduction (lines 13-18, 47-51, 57-61, 72-82). We also moved the material in the Results on "Increased coverage of catabolism in diverse bacteria and archaea" (lines 575 on) into its own section, to highlight the broader relevance of the gaps that we filled.

2. We redid Figure 1 (the 62 carbon sources described by GapMind) to not rely on color. Also we reclassified some sugar acids from “other” into "simple sugars."

3. GapMind and archaea. In the Results, we report how many of the characterized proteins in our database are from archaea, bacteria, or eukaryotes (lines 114-115). And in Figure 9C (on the quality of GapMind’s results), we now show GapMind's coverage of archaea separately. It is similar to GapMind's coverage for less-studied bacterial phyla.

4. Performance of GapMind's thresholds for high confidence hits. A new section in the Methods estimates the expected accuracy of GapMind's "high confidence" candidates (lines 786 on). This is briefly summarized in the Results: "Similarity near this threshold (40-50% identity and 80% coverage) corresponds to an estimated 73% accuracy for annotating enzymes, but just 32% accuracy for annotating transporters (see Methods)." The new section in the Methods also explains why we use a lax E-value threshold.

5. Figure 3 (role of NagX in glucosamine utilization) now shows controls (fitness data from glucose or lactate).

6. We report additional statistics as to the specificity of the mutant phenotype of the 3'-ketolactose hydrolase CCNA_01705 (lines 326-329).

7. We explain why, when more than one high-confidence path is present, GapMind prefers the longer one. "Our intuition is that one step might be annotated erroneously, but the presence of several steps is unlikely unless the pathway is present" (lines 561-563).

8. We clarify that GapMind examines only the predicted protein sequences and that ublast is a fast alternative to protein BLAST (lines 131-132 in the Results). Also, in the Methods, we explain that for genomes relating to the IJSEM database, we obtained the predicted protein-coding genes from RefSeq (line 671).

9. We clarify that models from TIGRFam are used if they are available for that enzyme classification (EC) number (lines 732-734).

10. To improve the readability of the fitness value in the heatmaps, we changed black text to white for values of -2 or less (which have a dark blue background). This affects Figures 3, 4, 6, and 7.

11. We reworded the relationship between the comparative genomics evidence for DUF2090 and our prediction for its function (lines 312-313).

12. We reorganized the discussion of the utilization of the 3-ketogalactose intermediate of lactose catabolism (line 362 and 401).

13. We reworded the description of the aerobic pathway for phenylacetate utilization (lines 407-411).

14. More detailed captions for Figures 4 and 6 and for supplementary figures 1-3.

15. We realized that the numbers of bacteria and archaea from IJSEM we reported analyzing were not correct. In our original submission, we counted some bacteria and archaea that are in IJSEM and have genome sequences, but are not reported to use any of the compounds in GapMind, so they were not included in the analysis in Figure 9C. The correct numbers are 224 bacteria and 13 archaea (line 670).

Responses to comments from reviewer #1

"... GapMind tool uses the PaperBLAST tool (which searches open-access scientific articles) to compare the genome with known sequences"

Although we used PaperBLAST to improve the definition of some steps, GapMind itself does not rely on searching scientific articles. Rather, GapMind uses ublast to compare predicted proteins to characterized proteins from the curated databases listed in Table 1. Also see change #8.

"...the lack of verification or analysis for archaeal species, which need be addressed before this work can be considered for publication"

During the curation of pathways and steps, we made every effort to include archaeal pathways in GapMind, and 6% of the characterized proteins in GapMind are from archaea (change #3). The revised Figure 9C shows that the coverage for archaea is a bit lower than for less-studied bacterial phyla (change #3). We believe that this reflects our limited knowledge of archaeal metabolism, rather than any problem with GapMind per se.

"Match thresholds. The thresholds for high, low, and medium confidence appear arbitrary, yet are important to the results of the GapMind tool. Please provide a justification..."

We added a new section in the Methods, titled "Thresholds for high-confidence candidates", to address this (change #4).

"2) Spurious alignments. The chosen E-value cut-off is 0.001, which is probably too high. Please justify why this value was selected"

The e-value cutoff was not mentioned in our original submission; it is 0.01. In principle, E < 0.01 is a lax threshold, but GapMind also requires identity >= 40% (or above 30% for medium confidence). In practice this is a more stringent threshold. We believe that it is better to use thresholds on the %identity and the %coverage, rather than on the e-value, because at a given %identity, log(E) scales with the alignment length, and some enzymes are much smaller than others. This is briefly discussed in the new section in the Methods on GapMind's thresholds (change #4).

"Additionally, for the alignments made to represent transcriptional/translational units, open reading frames need to first be determined, but this is not addressed in the text. Can the authors please elaborate on how they determined open reading frames?"

GapMind relies on the annotated protein-coding genes (change #8). Alternatively, the user can provide the 6-frame translation of the genome.

3) Fitness to analyze archaea genomes and catabolic pathways. GapMind is designed to be used for bacteria and archea; however, these are two different domains of life and can be very different in metabolism, enzymes, and enzyme sequences. Yet, only bacteria were extensively examined in this paper (save for a brief analysis reported in Figure 9C). In this analysis, it was found that a relatively low fraction of carbon sources used by archaea (about 20%) were marked as high confidence pathways. What is the justification for this tools fitness when applied to archaea?"

See above comment on archaea and change #3.

"Some potential justification could be given in Table 1 if there was a breakdown of the domain sources of the proteins used by GapMind (e.g., what fraction of these proteins are archaeal and bacterial), though this would not be sufficient in and of itself to address this concern. ...

About 6% of the characterized proteins that are associated with steps in GapMind are from archaea (change #3).

"This lack of training [for archaea] is not addressed in the conclusion to this manuscript when plans to incorporate more bacterial fitness data is discussed."

We agree that large-scale fitness data from archaea would be very informative. But, to our knowledge, no one has ever collected mutant fitness data across many conditions for any archaea. In contrast, collecting fitness data from more bacteria is straightforward.

"4) Approach to identifying catabolic enzymes. The approach to identifying catabolic enzymes taken by the GapMind tool is to identify catabolic pathways via blast analysis. However, even high-identity blast results do not necessarily mean that the sequence maps to that enzyme function (consider the results reported in figures 8A and 8B of this manuscript, particularly the high error rate at under 60% identity). Perhaps more important than whole enzyme identity is the identity of critical residues for enzyme structure and active site. ... As written, the manuscript then seems to indicate that residue identity is the most important factor in whether or not a protein has the function of a particular enzyme. The question of this concern is why is identity chosen as more important than conservation of important residues or HMMs?"

We agree that the active site and the specificity-determining residues determine a protein's function. But, for the vast majority of characterized enzymes and transporters, these residues are not known, or there are not enough characterized members of the family to interpret any changes to these residues. For instance, at an early point during the development of GapMind, we considered using the Structure Function Linkage Database, but just 1% of the reactions in GapMind at that time were represented in the SFLD.

The reviewer also estimated a high error rate based on figure 8, but that figure only shows proteins that we chose to reannotate or curate, so it might be biased. Also it includes transporters (which are more difficult to annotate) as well as enzymes. Our revision includes a direct estimate of the accuracy of annotating enzymes and transporters (change #4).

"This is something that might be brought to light using Hidden Markov Models (HMMs). While "candidates are identified by using ublast or HMMer" (line 125), the role of these techniques in identifying a list of catabolic or transport candidate enzymes is unexplained."

For enzymatic steps, GapMind uses HMMs from TIGRFam if available (change #9). More precisely, GapMind uses HMMs for a step if any of the models in TIGRFam are annotated with that EC number.

"5) Use of fitness data. In this work, fitness data was used both to improve – line 54 –tool and to support its predictions – line 69 - of the GapMind i.e., both for training and validation. Then how does the tool perform using data on which it is not trained?"

As stated in our manuscript, "the results for the bacteria that have fitness data show the best-case performance." Figure 9C shows the performance of GapMind on diverse bacteria and archaea (that it was not trained on).

"Would there be a prerequisite to train the tool to the data it is to analyze? These results seemingly describe a confirmation of the results this tool was designed to show. Can the authors address this issue in their discussion?"

As shown in Figure 9C, we tested GapMind on diverse bacteria and archaea that were not used to develop the tool.

"a. In the “About GapMind” section, it is stated that the tool uses “ublast” but when one clicks on the various links of a high- or medium- confidence result, they are given PaperBLAST reports. The web tool does not make it clear as to what “ublast” is and what it is used for. Is this a separate tool from PaperBLAST, or merely some interface which PaperBlast is performed through?"

PaperBLAST is *not* used to identify candidates; rather it gives additional information, such as homologs that are discussed in papers and might not be in any of the curated databases that GapMind relies on. We also added clarifying text to this web page.

"b. For some low-confidence results, there are no reported BLAST results. However, by the definition of “low confidence” which is given in the interface, there must be a blast hit with at least 50% coverage to be reported as “low confidence”. Where might this blast hit be found or recovered?"

GapMind considers a step to be low-confidence even if it has no candidates at all. We updated the web site so that steps with no candidates have this explanation.

"Further, while high- and medium- confidence matches will provide a link to pfam (as an added layer to evaluate the strength of the match), this would be most useful for low-confidence matches such as shown in the screenshot, yet because the blast result is not reported, the pfam analysis could not also be performed."

We feel that PFams are usually too broad to be useful for finding a specific transporter or an enzyme that acts on a specific substrate. The page for an individual candidate does include a link to the Conserved Domain Database, which can be used to check if the candidate has the appropriate domains.

"c. The caveats described in the results and discussion section “overview of GapMind for carbon sources” should be mentioned somewhere on the report page (for instance after the list of the carbon catabolic steps) so it is clear to users what GapMind does not do."

The "About GapMind" section of each page mentions various reasons why there are gaps (even though the organism performs the function), and that GapMind does not search the six-frame translation. Also, it includes a link to our preprint.

"d. For the blast reports, many would be interested not only in identity, but e-value (as this is a common metric used for the quality of a match) and percent positives (that is, amino acids substituted with similar-type amino acids)."

We feel that %identity is more useful than E values -- for a given level of similarity, log(E value) is proportionate to the alignment length (change #4). The page does show the bit score, which

is directly related to the E value, and we updated the web page that shows candidates to explain that 44 bits corresponds to E = 0.001.

"e. Once the report is generated, having website links like “home”, “return to results”, and similar webpage navigation tools would make the webpage easier to navigate once the results have been produced."

The banner at the top links to the main GapMind page, and the "Links" section at the bottom includes other navigation options.

"f. When looking at the PaperBLAST results, it is initially difficult to find the query sequence used (because it is located near, but not at, the bottom of the results page)."

Good point. The sequence is now available at the top of the PaperBLAST results, with one click.

"2) Transporter ambiguity. How does GapMind treat transporters which are annotated to transport several different substrates or are ambiguous about substrates transported (for instance, the gene Cthe_1862 for Clostridium thermocellum which is annotated as “multiple sugar transport system ATP-binding protein”)."

In GapMind, the same protein sequence can be known to perform many different steps, such as the transport of several compounds. For the specific case of Cthe_1862 -- this protein does not appear in GapMind's database of characterized proteins. It is a high-confidence candidate for MalK_aa (maltose ABC transporter, ATPase component) because it is 66% identical to MsmX (P94360), which is listed as a component of a maltose transporter in TCDB. MsmX is probably involved in uptake of other saccharides such as melibiose, but those compounds are not among the 62 represented in GapMind, so that is not currently relevant to GapMind.

"Is it permissive (e.g., assuming such a system could transport anything marked as a sugar) or strict (e.g., ignoring this gene as it does not specify exact substrates)?"

Imagine a hypothetical transporter that acts on both lactose and maltose. It would be listed as both a lactose transporter and a maltose transporter. Next, suppose that a similar protein is reported to transport maltose; it's not known if it transports lactose or not, but it's plausible. Ideally, we would mark this protein as "ignore" when identifying lactose transporters, so that similarity to this protein would not "count against" a candidate when assessing the confidence of candidates. ("Ignoring" similar enzymes is discussed in the Methods, in relation to the clustering tool.)

"3) Association between proteins. Please elaborate on why all the proteins discussed in the text are assumed to be distantly-related (e.g. divergent evolution from a single origin) as opposed to different events, such as convergent evolution. Is this true for each protein instance that is described?"

The diverged proteins have recognizable homology (i.e., using protein BLAST or PFam) to other proteins with similar functions. It is widely accepted that these superfamilies reflect divergence from a common ancestor (i.e., "Divergence and Convergence in Enzyme Evolution", PMID:22069324). "Convergent evolution" may refer to independent evolution of the same function within a family (say, within a superfamily of hexose transporters, two subfamilies become ribose transporters instead). We are not making any claims about convergent evolution or not (nor do we use terms like "divergent" or "convergent").

"4) L57: Please clarify what is meant by “initial version” of GapMind? Does that refer to an iterative process or to a prototype of the algorithm created?"

Meaning, that we created an initial version of GapMind without examining the fitness data, and then we used that data to improve the description of the pathways or steps in GapMind. We revised this sentence of the Introduction (as part of change #1).

"5) L69: Please clarify what is meant by “… GapMind usually selects the correct pathway…”? Was the success rate of GapMind estimated? If so, why isn’t it referred to explicitly?"

We did not make a precise estimate of how often GapMind selects the correct pathway. But in the section of the Results on "Quality of GapMind’s results", we reported that 82% of non-redundant enzymes on the best path were important for fitness (if we had data for that gene and condition), and we checked a random sample of 20 of the exceptions. For 6/20 cases, other steps in the pathway were important for fitness, so GapMind probably selected the correct path; in another 12/20 of those cases, there seems to be more than 1 path operating. Overall we think the statement, in the Introduction, that GapMind usually finds the best path, is justified, but the details are too complicated to explain there.

"6) L300-302: Bioinformatics evidence supports the authors’ proposition that DUF2090 is a 2-deoxy-5-keto-D-gluconate 6-phosphate aldolase but does not confirm it. Experimental evidence would be necessary to confirm such a proposition."

We agree. As the manuscript states, the bioinformatics evidence supports our prediction (but it's still a prediction). We hope the revised text (change #11) makes it more clear that the comparative genomics shows that DUF2090 is associated with inositol utilization (which is suggestive but does not prove anything about its role).

7) L541: Please justify why GapMind chooses the pathway with more steps, when that could possibly represent a more burdensome pathway for the cell to execute the same function?

Most catabolic enzymes are specific: they're not part of central metabolism or other pathways. So, the greater the number of genes present, the more likely that the pathway truly is present (change #7).

Also, the reviewer implied that pathways with more steps are more burdensome, but actually, the major burden is probably the amount of protein required, rather than the number of genes (i.e., "Glycolytic strategy as a tradeoff between energy yield and protein cost", PMID:23630264).

Responses to comments from reviewer #2

"- Novelty & framing of the paper's key contributions. The paper is set up (given title and abstract) as the description of a new tool (GapMind) for predicting catabolic pathways given a genome. However, it's unclear how this database/tool is different than the previously published GapMind resource cited in the manuscript (Price et al. mSystems 2020) other than the focus on carbon catabolism vs. amino acid synthesis.

Yes, the executable code is little changed, but a lot of effort went into curating all the pathways and steps.

"In addition, the manuscript discriminates between the annotation of catabolic pathways and the network model-derived predictions of the catabolic functions of an organism, arguing that predicting a catabolic function is difficult because of variance in protein expression or perhaps because of erroneous annotations. However, again, working through the differences between annotating catabolic pathways and model-driven predictions of catabolic functions is not really the focus of the paper. The real contribution of the paper seems to be how the use of the GapMind database can help provide context to the large mutant growth screening data to enable stronger predictions for functional annotation of catabolic pathways. This objective is a nice focus, the authors make a nice contribution on this point, and the new biology discussed as an outcome of these analyses is a nice contribution. All this to say that the manuscript should be revised to better frame these more valuable contributions of the paper."

We changed the title and revised the abstract and introduction to try and follow this suggestion (change #1).

- GapMind identifies a path with "all high-confidence steps" or that has "no low-confidence steps", or that has the "highest total score". The manuscript would benefit from an analysis of the differences between these thresholds and sensitivity to all these types of thresholds that are used in assigning confidences and in "selecting" a pathway.

As shown in "Quality of GapMind's results," most of the non-redundant enzymes on the best path are important for fitness, which implies that GapMind selects the correct pathway most of the time. Furthermore, most of the exceptions seem to be due to there being more than one pathway; arguably, given only the genome sequence, there's no right answer in those cases.

- The reference pathways included in the GapMind database include those for which a complete pathway is known or for which one step is missing. The robustness of the analysis in the paper to this decision should be considered. What happens if pathways are included with two missing steps? Zero missing steps?

GapMind includes every catabolic pathway for these 62 compounds that we are aware of that is reported to occur in bacteria or archaea and is fully known (that is, each enzyme is linked to sequence, so there are zero missing steps). We don't think it makes sense to represent pathways with several missing steps, as the gaps would be impossible to assess. We might be wrong about this, but testing it would be a significant effort, as it requires curating every step from the incomplete pathways. We don't expect this effort to be worth it. The "Curating pathways" section of the manuscript does list some of the poorly-understood pathways that we are aware of.

- For the last section of the Results on assessing the quality of GapMind's results, it is unclear what is actually being assessed. It seems the validation is grow/no grow data of 29 bacteria on 57 carbon sources, but a key component of GapMind predictions are which pathways are used for a given catabolic function and the grow/no grow data the authors reference do not seem to have that kind of resolution.

Besides looking at growth/no growth, we also asked whether the genes on the best path were important for fitness (Figure 9B).

"While there are many new annotation assignments presented in this manuscript, it almost becomes a little unwieldy and the manuscript would benefit from a more concise summary and more focused description of fewer specific stories."

We have a separate section for each novel pathway or novel enzyme family, and an overview section for the more diverged (and hence more interesting) proteins ("Annotation of 299 diverged enzymes and transporters"). If the editor requests, we can move some of the novel enzyme families to the supplementary material.

"The black text on the dark blue background in several figures is difficult to see."

This has been changed (change #10)

"There are a couple instances where the referenced data is from "personal communications" [in reference to whether all data underlying the figures and results presented in the manuscript has been provided]

The fitness data from Paraburkholderia bryophila 376MFSha3.1 with phenylacetate as the carbon source, or from Pseudomonas putida KT2440 with glucuronate or galacturonate as the carbon source, are available, both in the Fitness Browser

https://fit.genomics.lbl.gov/cgi-bin/exps.cgi?orgId=Burk376&query=phenylacetic&Go=Go

https://fit.genomics.lbl.gov/cgi-bin/exps.cgi?orgId=Putida&query=%25uronic&Go=Go

and in the figshare. These data are referenced with "personal communication" because we did not collect them.

The fitness data for Ga0395975_5191 from Burkholderia sp. OAS925 is not publically available (and, again, we did not perform that experiment). We only mention it briefly to confirm the results for H281DRAFT_04594 from Paraburkholderia bryophila. If necessary, this could be removed from the manuscript.

Responses to comments from reviewer #3

"1. Inclusion of controls a. For the role of glucosamine utilization (Page 10 to Page 12), the investigators have not used any control. Even though the analysis is good, inclusion of a substrate control such as glucose would strongly support the conclusions."

We added data from glucose or lactate to each panel in Figure 3 (change #5)

"2. Figures can be improved. a. There are fitness assay results that use one or two experiments. In such cases, taking averages might not make sense. But there are many instances where the authors decided to report individual numbers. Despite being more informative, the figures do not look engaging. I suggest revision of the visualization especially for fitness data."

Almost all of the figures show replicate experiments separately (usually 2-3 replicates, although there are exceptions). We think this is the best way to show the data because it allows the viewer to check how consistent the data is.

"b. Resolution of some of the figures is poor. If possible, they should be improved."

We think this was an artifact of the process of producing a PDF; for the revision we uploaded the figures separately and this should be resolved.

"1. Improvements in the figures a. Figure 1 (Page 6): Visually it can be better. I would also modify the color scheme (especially changing green or red)."

We redid this figure (change #2)

"b. Figure 6 (Page 20): The grey rows are not properly explained in the figure caption. If the data is absent for the gene, these rows can be entirely removed."

We updated the caption to explain that grey means no data (change #14). We thought it best to keep those rows. In panel A, the row is the only indication that glucose 6-phosphate dehydrogenase is in the cluster of glucose utilization genes. And in panel C, we'd have to explain why lacB was not shown, so it seemed simpler to leave it in.

"c. Figure 4 (Page 14): i. The figure caption can be improved (especially for figure E). ii. CtlX should be ctlX as it is a gene, and not a protein in this context."

We corrected the caption for 4D and added more detail to the caption for 4E (change #14).

"d. Supplementary figures: Figure captions can be improved."

We added more detailed captions (change #14).

"2. Quantitative tests for determination of differences a. For the experiments involving ketolactose hydrolase, statistical tests for comparison between different substrates (for gene fitness) would be better."

Across 198 fitness assays with a wide range of conditions, the putative ketolactose hydrolase (CCNA_01705) has a strong fitness defect (value of -2 or less) only during growth on lactose (3/3 replicates) or raffinose (1/3 replicates). We added a brief mention to the text (change #6).

"3. Line 386 to 389 (page 23): This information needs further elaboration as I did not understand why authors chose to bring up 3-ketohexose/3-ketoglucose utilization."

We moved some of this material to an earlier paragraph and we hope it is clearer now (change #12). First, 3-ketohexose utilization might explain the role of some of the other members of the lactose oxidation gene cluster. Second, it's important to explain that the later steps in the pathway are not known. Finally, since the pathway is not fully understood, we thought we should explain how it is represented in GapMind.

"4. Line 393 to 397 (page 23): The sentence should be rephrased as it appears to be grammatically inconsistent."

We rewrote this sentence (change #13).

"5. Line 540 to 542 (page 31): The authors suggest that if two high confidence pathways are identified, the tool arbitrarily chooses the one with more steps. This is counterintuitive to metabolic pathway algorithms that use parsimonious approach.

See change #7.

"If a minimum number of steps approach is taken instead, will accuracy be affected negatively?"

Yes, but mostly for transporters, which probably wasn't what the reviewer had in mind. In more detail: we reran GapMind on the organisms with fitness data, with the ranking of high-confidence pathways modified to prefer the shortest pathway. We then checked the fitness data for genes that were on the longest high-confidence path, but not on the shortest one, or vice versa. We considered only non-redundant genes (there was only one high-confidence

candidate for that step) that had fitness data. For enzymes, we found that enzymes on the longest pathway were about as likely to be important for fitness as those on the shortest pathway (41% vs. 38%, $P > 0.5$, Fisher exact test). These proportions are much lower than for enzymes overall (82%), which suggests that many of these pathways really are redundant. In contrast, for transporters, the longest-path genes were much more likely to be important for fitness (37% vs. 19%, $P = 1.4e-5$, Fisher exact test). It appears that annotations of multi-subunit transporters are more reliable. This is consistent with our intuition (change #7).
